# Iatrogenic Penile Glans Amputation: Major Novel Reconstructive Procedure

**DOI:** 10.1155/2013/741980

**Published:** 2013-11-27

**Authors:** Rami Nasr, Samer L. Traboulsi, Rami R. Abou Ghaida, Joseph Bakhach

**Affiliations:** ^1^Division of Urology, Surgery Department, American University of Beirut, P.O. Box 11-0236 Riad El Solh, Beirut 1107 2020, Lebanon; ^2^Division of Plastic Surgery, Surgery Department, American University of Beirut, P.O. Box 11-0236 Riad El Solh, Beirut 1107 2020, Lebanon

## Abstract

Circumcision is a very common urological practice. Even though it is relatively safe, it is not a complication-free procedure. We describe a patient that underwent a neonatal circumcision complicated by iatrogenic complete glans amputation. Reconstructive repair of a neoglans using a modified traditional method was used. Postoperative followup to 90 days is illustrated. Despite being a simple procedure, circumcision in unprofessional hands can have major complication impacting the emotional and sexual life of patients. Surgical reconstruction is possible with varying satisfactory results.

## 1. Introduction 

Circumcision is one of the most common operations performed in urology. It is usually a safe and simple procedure with a very low morbidity rate; however, serious complications can occur [1]. In this part of the world, circumcision is regarded as a religious ritual. It is being practiced widely by nonmedical personnel in many rural areas. Due to this unprofessional practice, many complications arise ranging from infections to disfigurement or partial amputation of the penis. The true incidence of complications is unknown because of underreporting and because the majorities are small insignificant iatrogenic injuries that are managed by basic medical first aid treatment and wound pressure and that rarely require surgical intervention. Gee and Ansell reported a complication rate of 0.2–0.6%, ranging from bleeding, lymphedema, fistula formation, iatrogenic hypospadias to the most serious one being amputation of the glans penis [2, 3]. Most amputations are not documented as they are repaired immediately. The most dreadful complication is partial or complete glans amputation. If recognized within 8 hours, the glans can be successfully reattached [4]. Penile glans reconstruction due to partial or complete injury is a challenging issue for both urologists and plastic surgeons and the aim of the reconstruction is to provide an esthetically acceptable shape, allow for normal or near normal intercourse, preserve sensation if possible, and perform a surgery that minimizes the risks of fistulae or urethral strictures.

## 2. Case

Our patient is a 35-year-old healthy single male. His only concern was penile size. He had a neonatal circumcision performed by a surgeon but was complicated by bleeding. He was sexually active; however, the psychological impact of genital disfigurement was present.

**Figure 1:**
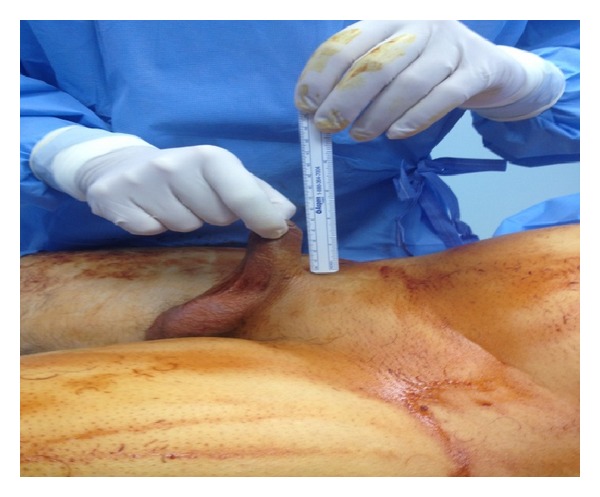
Short penile stump.

On physical examination, the stretched penile size was 6 cm with scar tissue at the tip (Figure 1), a stenotic urethral meatus, and no obvious glans tissue present. An MRI was done to assess the corpora cavernosa and the extent of injury. There was no anatomic glans and the corpora cavernosa had decreased enhancement due to fibrosis. After counseling the patient, we elected to use the technique of neoglans reconstruction described by Shaeer and Sebaie in 2005 [5] with a few modifications.

## 3. Description of the Procedure 

**Figure 2:**
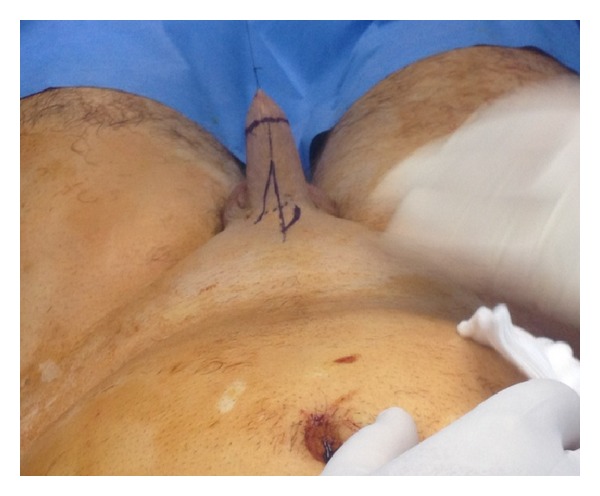
Dorsal and ventral Z plasties done.

**Figure 3:**
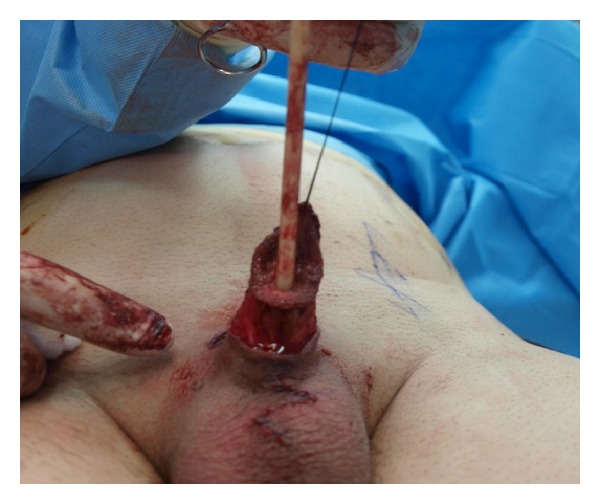
The distal penile skin dissected.

**Figure 4:**
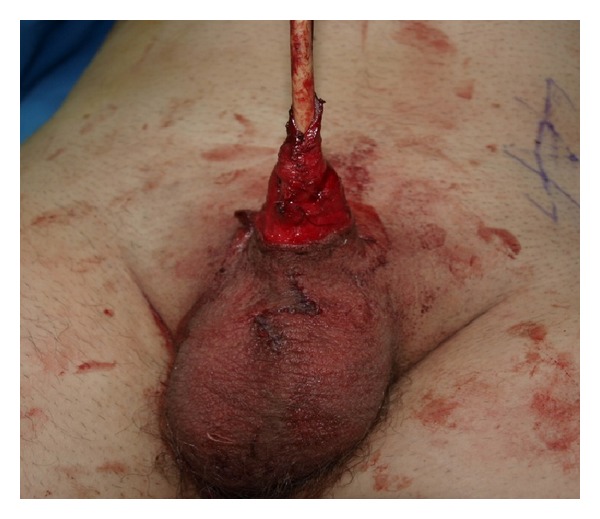
The distal skin tubularised over 14 Fr catheters.

**Figure 5:**
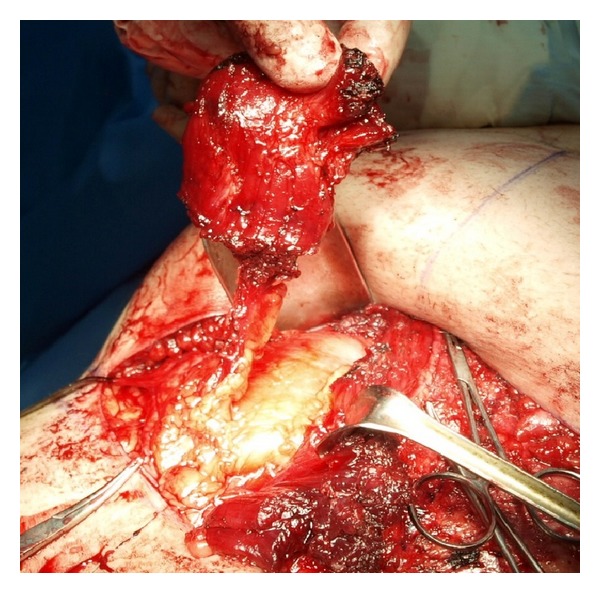
The rectus muscle flap with the distal penile shaft.

**Figure 6:**
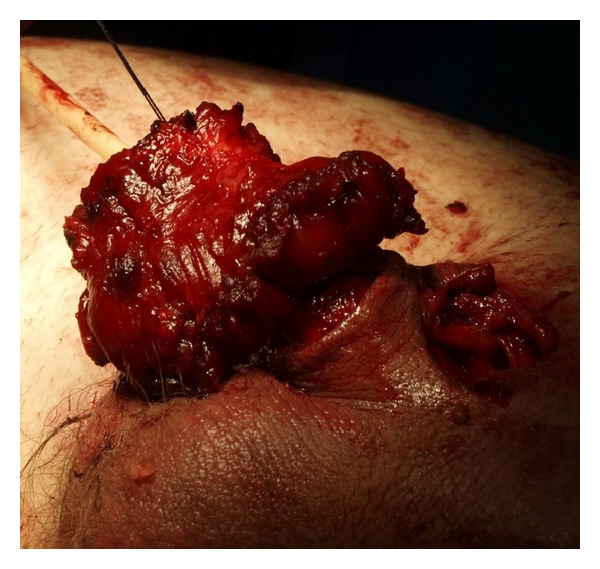
The flap being sutured to the distal penile neurovascular bundle.

The patient was put in the supine position and the whole abdomen and genitalia till the lower thighs were scrubbed and draped. A Z-plasty incision was done dorsally and ventrally at the base of the penis (Figure 2), and dissection of the corpora cavernosa from the inferior pubic bone was done (Figure 3). The dorsal vein was ligated, the suspensory ligament was cut, the corpora were sutured to each other, and the suspensory ligament was reattached to a more proximal part of tunica taking care not to injure the neurovascular bundle. To give more length to the stump, we performed Liposuction of the mons pubis and the penile base. The scar tissue at the tip of penile stump was dissected and (as done by Shaeer et al.) a neourethra was fashioned by tubularizing the distally degloved foreskin over a 14 Fr foley catheter (Figure 4). Through a Pfannenstiel incision, a rectus abdominis flap of 4 × 12 cm was harvested with preservation of the deep epigastric neurovascular supply (Figure 5) and was tunneled under the skin of the suprapubic area and the penile shaft skin to reach the area of the neoglans (Figure 6). The flap was covered with a split thickness skin graft harvested from the hairless left anterior thigh.

**Figure 7:**
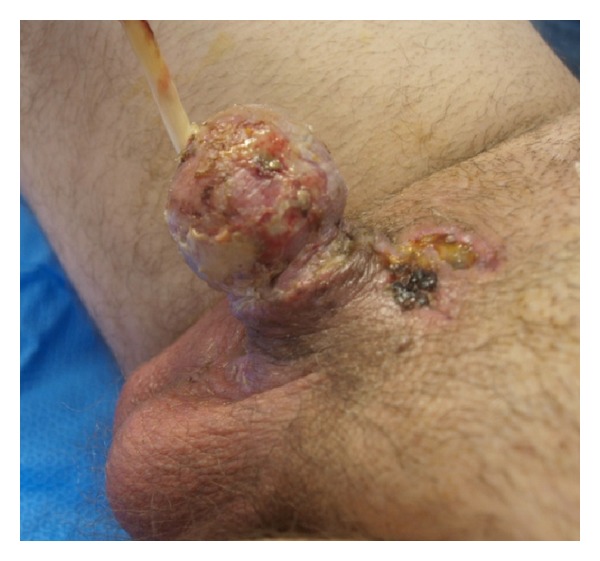
One week followup.

**Figure 8:**
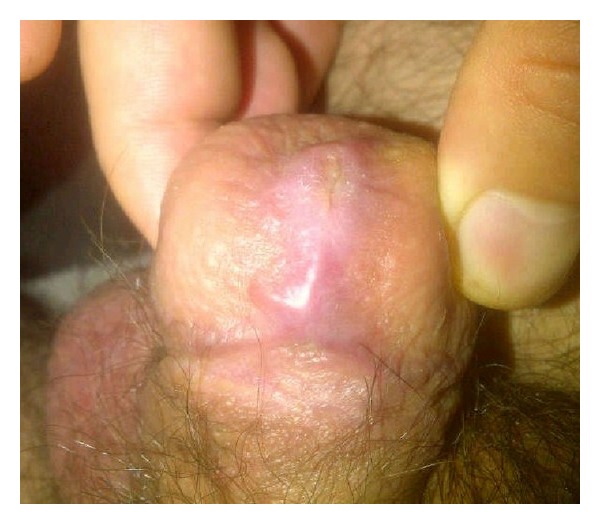
Glans at six weeks followup.

On one week follow up, a small fistula occurred at the base of the ventral anastomosis but it closed spontaneously after dilating the meatus (Figure 7). The glans penis split thickness graft was derided at three weeks with the graft looking very healthy. At followup after six weeks, the glans looked healthy and had shrunken by about 40% (Figure 8).

## 4. Discussion 

Glans amputation is still the most serious complication of neonatal circumcision. It can be partial or complete for various reasons, that is, traumatic, self-mutilation or transgender exchange, or after amputations for penile cancer. If the injury is diagnosed during the procedure, the stump can be reattached even after eight hours with good cosmetic and functional results [4, 6]. There are many techniques for glans reconstruction after complete or near complete amputation.

Whenever possible, the glans should be reconfigured from its native tissue, but many times we are faced with the situation in which we have complete amputation and there is a need for neoglans reconstruction [7]. Belinky et al. used the distal urethra to cover the corpus cavernosal tissue, but this needs healthy urethra and a fairly long penile stump to have an acceptable sexual and cosmetic result [8]. Mazza and Cheliz developed a two-stage technique in which a scrotal fasciocutaneous flap is tubularized and sutured to the distal end of the penis. The flap pedicle is then resected under local anesthesia after six weeks. This method provides good cosmesis but at the expense of a two-stage operation and a high rate of meatal stenosis [9]. Others used buccal mucosa to cover the distal ends of the cavernous tissue after penile cancer surgery, but this graft tends to have a high rate of contractures [10].

**Figure 9:**
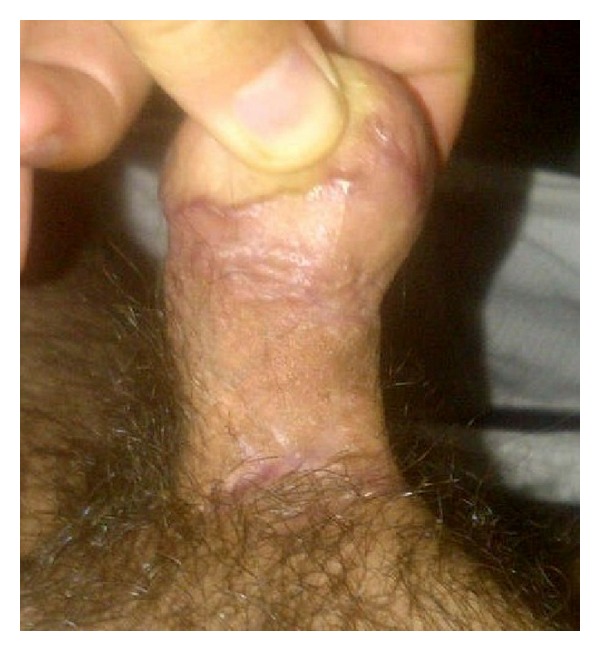
Ninety-day followup.

We chose the method of Shaeer and Sebaie [5] because it is rather simple to perform, is a one stage procedure, and provides acceptable cosmetic and functional results. Our patient had an MRI for the penis with gadolinium to assess the extent of injury that showed acceptable residual length of the corpus-cavernous bodies. The rectus abdominis flap purpose is to give the shape of the neoglans and to provide few centimeters to the overall penile length. We performed few maneuvers to add more length including dorsal and ventral Z plasties, cutting the suspensory ligaments, and performing liposuction of the pubic area. Thus four centimeters were added to the shaft. The patient did well and was satisfied regarding cosmesis at ninety-day followup (Figure 9). However, he developed meatal stenosis and a small fistula at the ventral side of the flap that eventually closed after being instructed to perform self-meatal dilatation twice daily.

A potential problem of this technique is mainly fistula formation. In order to avoid fistulas like the one we encountered, we suggest rotating the flap to the sides so that the suture lines do not overlap. Functionality regarding sexual activity after such a procedure still has to be evaluated. Other less likely complications of this technique are flap necrosis, avoided by carful harvesting and ensuring adequate blood supply to the graft. Infection is minimized by using antibiotics with skin flora and antipseudomonas coverage.

## 5. Conclusion 

Circumcision is a regarded as a minor surgical procedure; however, it is not free of complications. Complications can have lifetime functional, psychological, and cosmetic sequel. There are many techniques described in the literature, and in our opinion, the best corrective procedure would be a one stage intervention. Most of the techniques are partially satisfactory; therefore, prevention of this complication is the best treatment.
